# Phase II trial of utidelone as monotherapy or in combination with capecitabine in heavily pretreated metastatic breast cancer patients

**DOI:** 10.1186/s13045-016-0297-7

**Published:** 2016-08-11

**Authors:** Pin Zhang, Zhongsheng Tong, Fuguo Tian, Yongsheng Wang, Junlan Yang, Weilian Li, Lijun Di, Wei Liu, Li Tang, Rongguo Qiu, Binghe Xu

**Affiliations:** 1 National Cancer Center/Cancer Hospital, Chinese Academy of Medical Sciences and Peking Union Medical College, 17# Panjiayuan Nanli, Beijing, 100021 China; 2 Cancer Hospital, Tianjin Medical University, Tianjin, China; 3 Shanxi Cancer Hospital, Taiyuan, Shanxi China; 4 Shandong Cancer Hospital, Jinan, Shandong China; 5 PLA General Hospital, Beijing, China; 6 Tianjin People’s Hospital, Tianjin, China; 7 Cancer Hospital, Peking University, Beijing, China; 8 Hebei Cancer Hospital, Shijiazhuang, Hebei China; 9 Beijing Biostar Technologies, Ltd., Beijing, China

**Keywords:** Utidelone, Metastatic breast cancer, Phase II trial, Drug resistance

## Abstract

**Background:**

The treatment of metastatic breast cancer (MBC) remains a great clinical challenge as drug resistance frequently develops. Alternative agents that can overcome drug resistance would offer new therapeutic options. The primary aim of this phase II study was to evaluate the efficacy and safety of utidelone as a monotherapy or in combination with capecitabine in metastatic breast cancer patients previously treated with and resistant to anthracyclines and taxanes.

**Methods:**

In two open-label, noncomparative clinical studies, patients with metastatic breast cancer who previously received anthracycline- and/or taxane-containing regimens were given (1) 25 to 35 mg/m^2^/day intravenously infused utidelone, once daily for 5 days, in combination with 14 days of 2000 mg/m^2^ capecitabine, divided in two equal daily oral doses or (2) 40 mg/m^2^/day intravenously infused utidelone, once daily for 5 days. These regimens were administered per each 21-day treatment cycle, and the maximum of treatment cycles allowed per protocol is 6. Objective response rate (ORR), progression-free survival (PFS), and tolerability were evaluated.

**Results:**

In the combination study, 33 patients completed a median of 6 cycles of therapy, which was the highest cycles a trial patient could receive under the criteria of the study protocol. Efficacy was evaluated (*n* = 32) with an ORR of 42.4 % (FAS, 95 % CI, 26.6, 60.9) and a median PFS of 7.9 (FAS, 95 % CI, 6.1, 9.8) months. The monotherapy study (*n* = 63) resulted in an ORR of 28.57 % (FAS, 95 % CI, 18.4, 40.6) and a median PFS of 5.4 (FAS, 95 % CI, 2.9, 9.8) months. In both studies, common toxicities associated with utidelone administration included peripheral neuropathy, fatigue, myalgia, and arthralgia, but the toxicities were limited and manageable. Notably, very mild myelosuppression, low liver and renal toxicities, and very limited gastrointestinal toxic effect were observed, in contrast to other agents in the same class.

**Conclusions:**

Utidelone showed promising efficacy, tolerability, and advantageous safety profiles in the treatment of patients with advanced anthracycline/taxane-refractory metastatic breast cancer and may offer new treatment options to overcome drug resistance.

**Trial registration:**

CHiCTR-TRC-13004205, registered on August 15, 2013.

## Background

Epothilones are a class of naturally existing molecules produced by the myxobacterium *Sorangium cellulosum*. Bollag et al. [1] first identified the potential antineoplastic activity of epothilones, with a similar mechanism of action to taxanes. Specifically, epothilone binds to the β-subunit of the αβ-tubulin heterodimer, induces the formation of microtubule bundles, stabilizes the microtubule assembly, and prevents their depolymerization. This inhibits the formation of mitotic spindles and disrupts cell mitosis and other cellular growth and repair mechanisms. As a result, epothilone suppresses tumor cell growth, causing cytotoxicity and apoptosis.

Despite the similar mechanism of action, the molecular structure of epothilones differs from that of taxanes. Thus, tumor cells resistant to taxanes remain sensitive to epothilones’ cytotoxic effects [2]. As resistance to taxanes becomes an increasing challenge in their clinical use, alternative agents that can overcome tumor drug resistance would offer new therapeutic options for patients whose tumors recur or progress after taxane treatment. In preclinical research, epothilone analogs have demonstrated more potent cytotoxic activity than taxanes and activity against tumor cell lines with multidrug resistance [3]. Ixabepilone (Ixempra®), a semi-synthetic epothilone analog, is the only drug in this class that has been approved by the United States Food and Drug Administration (FDA) as monotherapy to treat metastatic or locally advanced breast cancer patients after failure of an anthracycline, a taxane, and a capecitabine treatment [4]. The combination of ixabepilone plus capecitabine is indicated for the treatment of metastatic or locally advanced breast cancer in patients after failure of an anthracycline and a taxane [5].

Utidelone (UTD1) is an analog of epothilone generated by genetically manipulating the polyketide biosynthetic gene cluster in *S. cellulosum* [6]. This agent was developed and manufactured by Biostar Technologies, Ltd., Beijing, China. Previously, we had conducted a phase I and a phase Ib study [6] (and unpublished data); limited, short-term toxicities, and encouraging preliminary efficacy results were obtained. Especially in the phase Ib study, we tested 30, 35, and 40 mg/m^2^ three different utidelone doses, given intravenously once daily for 5 days per 21-day cycle, instead of single dosing every 21 days as in the phase I trial [6]. These previous studies showed that the continuous 5-day dosing regimen gave better efficacy (unpublished data) than the single dosing regimen [6]. The DLT of the 5-day dosing regimen of utidelone was peripheral neuropathy, and the MTD was 40 mg/m^2^/day (unpublished data). Based on these earlier studies, this phase II multicenter clinical trial was designed and initiated to evaluate the efficacy and safety of utidelone in heavily pretreated patients of metastatic breast cancer.

## Methods

### Patients

The patients were 18- to 70-year-old female patients who were histologically or cytologically diagnosed with advanced metastatic breast cancer and had received no more than three regimens of chemotherapies (the adjuvant therapy did not count as one regimen) in the past or experienced recurrence. The previous chemotherapy regimens must have included an anthracycline and/or a taxane (including neoadjuvant and adjuvant therapies), but no more than three chemotherapy regimens were given in the metastatic setting. Additional inclusion criteria included an Eastern Cooperative Oncology Group (ECOG) performance status of 0 to 2, life expectancy of at least 3 months, at least one target site that could be evaluated via imaging techniques, and nervous system disorders lower than grade 2 on the National Cancer Institute (NCI) Common Toxicity Criteria (CTC) version 4.03. Informed consent was obtained from each patient before initiating study procedures, and studies were performed in accordance with the appropriate locally governing institutional review board committee, formally designated to approve, monitor, and review biomedical research involving human study subjects.

### Study design and treatment

This research consisted of two open-label, noncomparative, phase II clinical studies of utidelone. The studies included a utidelone plus capecitabine combination study and a utidelone alone monotherapy study.

The combination therapy study was conducted in two stages. First, a dose escalation study was conducted at one clinical study site (*n* = 3–5 patients per dose) with intravenously infused utidelone administered once daily for 5 days, in combination with 1000 mg/m^2^ twice daily oral capecitabine (Hoffmann-La Roche AG, Basel, Switzerland) for 14 days in each 21-day treatment cycle. Three doses (25, 30, 35 mg/m^2^/day) were escalated; three to five patients per dose were treated. Based on the safety and efficacy results obtained from the three dose groups, the second stage of the study was expanded to three clinical study sites with the MTD dose (30 mg/m^2^/day) of utidelone, chosen for all subsequent study subjects to be administered with capecitabine. Eligible patients were continually enrolled into the study until the total number of study subjects reached the planned sample size of 21 patients. This sample size was determined according to a superiority design, *α* = 0.10, *β* = 0.80, using a phase III trial result of ixabepilone as a reference, which had an objective response rate (ORR) of 34 % for ixabepilone plus capecitabine, and an ORR of 14 % for capecitabine alone.

**Fig. 1:**
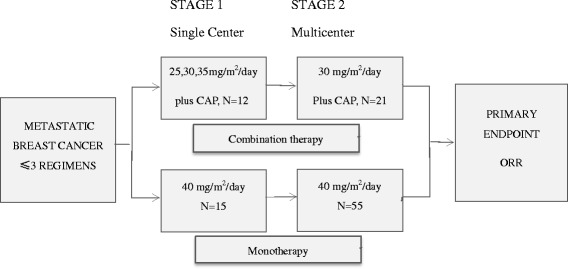
Study strategy and design. Patients previously received three or fewer chemotherapeutic regimens (not including adjuvant therapy) with MBC were enrolled. Both combination therapy and monotherapy were carried out in two stages. In the first stage of combination therapy, three doses of utidelone (30, 35, and 40 mg/m^2^/day) plus capecitabine (2000 mg/m^2^/day) were tested, with each dose group recruiting 3–5 patients of total 12 patients. Based on the efficacy and toxic effects of the combined agents, 30 mg/m^2^/day dosing regimen was selected, which demonstrated better safety profile with promising efficacy, as the dose for the second stage that recruited 21 patients. For the monotherapy, compare two different dosing regimens in the first stage: (1) 170 mg/m^2^ iv, once every 21 days; (2) 40 mg/m^2^ iv, once daily for 5 days every 21 days. Patients were randomized into the two different regimens, and 15 patients for each group were enrolled. Based on the efficacy and safety of the first stage, 40 mg/m^2^/day dosing regimen was chosen for the second stage to continue recruitment until target patient number reached 55. Efficacy evaluation was carried out once every 2 cycles, and the primary endpoint ORR was assessed

In the monotherapy study, eligible patients were given utidelone 40 mg/m^2^ daily for 5 days in each 21-day treatment cycle. In this trial, multicenter two-stage design was used with an ORR of 15 %, a null response rate of 5 %, a type one error of 0.10, and a type two error of 0.10. As such, continuation to the second stage required at least 1 patient out of the first 15 to demonstrate partial response (PR) or complete response (CR). Eligible patients were continually enrolled into the second stage until the total number of study subjects reached the planned sample size of 60 patients. The study strategy and design was illustrated in Fig. 1.

### Efficacy and tolerability assessments

The primary endpoint measured in both studies was ORR determined according to Response Evaluation Criteria in Solid Tumors (RECIST) version 1.1. The secondary end points included progression-free survival (PFS) and safety. Safety was monitored by investigator and laboratory tests.

Study subjects were evaluated for tumor response every two treatment cycles. Those with status of stable disease (SD), PR, or CR were eligible to continue the study treatment for up to six treatment cycles or until discontinuation due to disease progression or intolerable toxicity. After the end of treatment, post-monitoring of study subjects with SD or better efficacy was conducted every 3 months until disease progression or death. Study subjects who discontinued study treatment due to reasons other than progression were followed every 3 months thereafter until disease progression or death.

Routine hematologic laboratory tests were performed once weekly, between day 3 and day 5, until the completion or discontinuation of study treatment. Routine biochemistry laboratory tests were performed once every cycle between day 3 and day 5 in study week 3. In addition, ECG, vital signs, and physical examination were performed at regular intervals during the study for abnormalities and significant changes from baseline. All assessments and tests were performed at the baseline and end-of-treatment visits. Additional and more frequent tests could be ordered at the investigator’s discretion.

### Statistical analysis

The efficacy and safety variables were summarized using descriptive statistics, as no comparator was used in this research. Survival curves were estimated using the Kaplan-Meier method. For the efficacy variables (i.e., ORR, PFS), 95 % confidence intervals were calculated using the Clopper Pearson method.

## Results

### Study subjects and treatment cycle completion

The demographic information of patients for both monotherapy and combination therapy is summarized in Table 1. All study subjects were Chinese women with stage IV breast cancer whose ages ranged from 28 to 71 years (one patient is 71, over the age 70 inclusion criteria requirement, but a study deviation was permitted). The median age was 50.3 years in the combination therapy study and 50.9 years in the monotherapy study. Most study subjects had metastases to the liver and/or lung (>70 %). Over 80 % study subjects had previously received both taxanes and anthracyclines, and over 30 % had also received capecitabine.

**Table 1:** Study subject demographics

Demographic characteristic		Combination therapy study: utidelone (30 mg/m^2^) and capecitabine (2000 mg/m^2^) *n* = 33	Monotherapy study: utidelone (40 mg/m^2^) *n* = 70
Age (year)	Median	50	51
	Range	28–66	31–71
Sex, *n* (%)	Female	33 (100)	70 (100)
ECOG PS, *n* (%)	0	19 (57.6)	9 (12.9)
	1	14 (42.4)	60 (85.7)
	2	0 (0.0)	1 (1.4)
Clinical stage, *n* (%)	IV	33 (100)	70 (100)
Metastasis site, *n* (%)	≤2	20 (60.6)	47 (67.1)
	>2	13 (39.4)	23 (32.9)
Number of target lesions, *n* (%)	1	18 (54.5)	39 (55.7)
	2	10 (30.3)	18 (25.7)
	>2	5 (15.2)	13 (18.6)
Past chemotherapy courses, *n* (%)	1	6 (18.2)	10 (14.3)^a^
	2	12 (36.4)	20 (28.6)
	3 or more	15 (45.5)	40 (57.1)
Past treatment regimens containing, *n* (%)	Anthracyclines	33 (100)	67 (95.7)
	Taxanes	30 (90.9)	67 (95.7)
	Taxanes + anthracyclines	27 (81.8)	64 (91.4)
	Capecitabine	13 (39.4)	34 (48.6)
	Taxanes + anthracyclines + capecitabine	11 (33.3)	33 (47.1)
Concurrent diseases or complications, *n* (%)	No	30 (90.0)	54(77.1)
	Yes	3 (9.1)	16(22.9)
Primary tumor excised, *n* (%)	Yes	32(97.0)	68(97.1)
Received radiation therapy, *n* (%)	Yes	18(57.6)	48 (68.6)

*ECOG PS* Eastern Cooperative Oncology Group performance status

^a^Neoadjuvant and/or adjuvant chemotherapy

The study strategy and design was illustrated by Fig. 1.The first stage of combination therapy was carried out to determine an optimal dose regimen; 25, 30, or 35 mg/m^2^/day utidelone plus 2000 mg/m^2^/day capecitabine was conducted in one study site. Three patients were administered the 25-mg/m^2^/day dose, four were administered the 30-mg/m^2^/day dose, and five were administered the 35-mg/m^2^/day dose. The DLT of the combination was peripheral neuropathy and was observed at 35 mg/m^2^/day dose, and the MTD for utidelone was 30 mg/m^2^/day. Based on the results from the first stage of the combination therapy, the MTD dose 30 mg/m^2^/day with better efficacy and safety profile was chosen for the later enrolled patients, and the study was expanded to three study sites until the total number of study subjects reached the planned sample size of 21 patients for the 30-mg/m^2^/day dose group. A total of 33 patients with late-stage metastatic breast cancer were enrolled from July 2012 to April 2013. Eighteen of the 33 study subjects completed the maximal six treatment cycles specified in the protocol. Two study subjects requested and were allowed for one and two additional cycles of compassionate treatment after completing 6 cycles. The median number of treatment cycles completed was six, which was the highest cycles a trial patient could receive under the criteria of the study protocol.

The monotherapy study with 40 mg/m^2^/day utidelone was conducted at eight enrollment sites starting in August 2012. The last patient was enrolled in June 2014. A total of 70 study subjects with late-stage metastatic breast cancer were enrolled. Twenty of the 70 study subjects completed the maximal 6 cycles of treatment. Three study subjects requested and were allowed one to two additional treatment cycles of compassionate treatment after completing 6 cycles. The median number of treatment cycles completed for the monotherapy study was 3.5; 12.9 % required reduction of utidelone in the monotherapy study. The number of study subjects completed treatment cycles is summarized in Table 2.

**Table 2:** Study subject treatment cycles completed

	Combination therapy study: utidelone (30 mg/m^2^/day) + capecitabine (2000 mg/m^2^/day) *n* = 33	Monotherapy study: utidelone (40 mg/m^2^/day) *n* = 70
Utidelone dose	30 mg/m^2^/day for 5 days per cycle	40 mg/m^2^/day for 5 days per cycle
Cycles completed		
1	1	7
2	5	24
3	2	4
4	4	10
5	3	5
≥6^a^	18	20
Median	6	3.5
Mean	4.82	3.64

^a^Compassionate treatment for additional treatment beyond 6 cycles were allowed for some patients for their benefits

### Efficacy

**Fig. 2:**
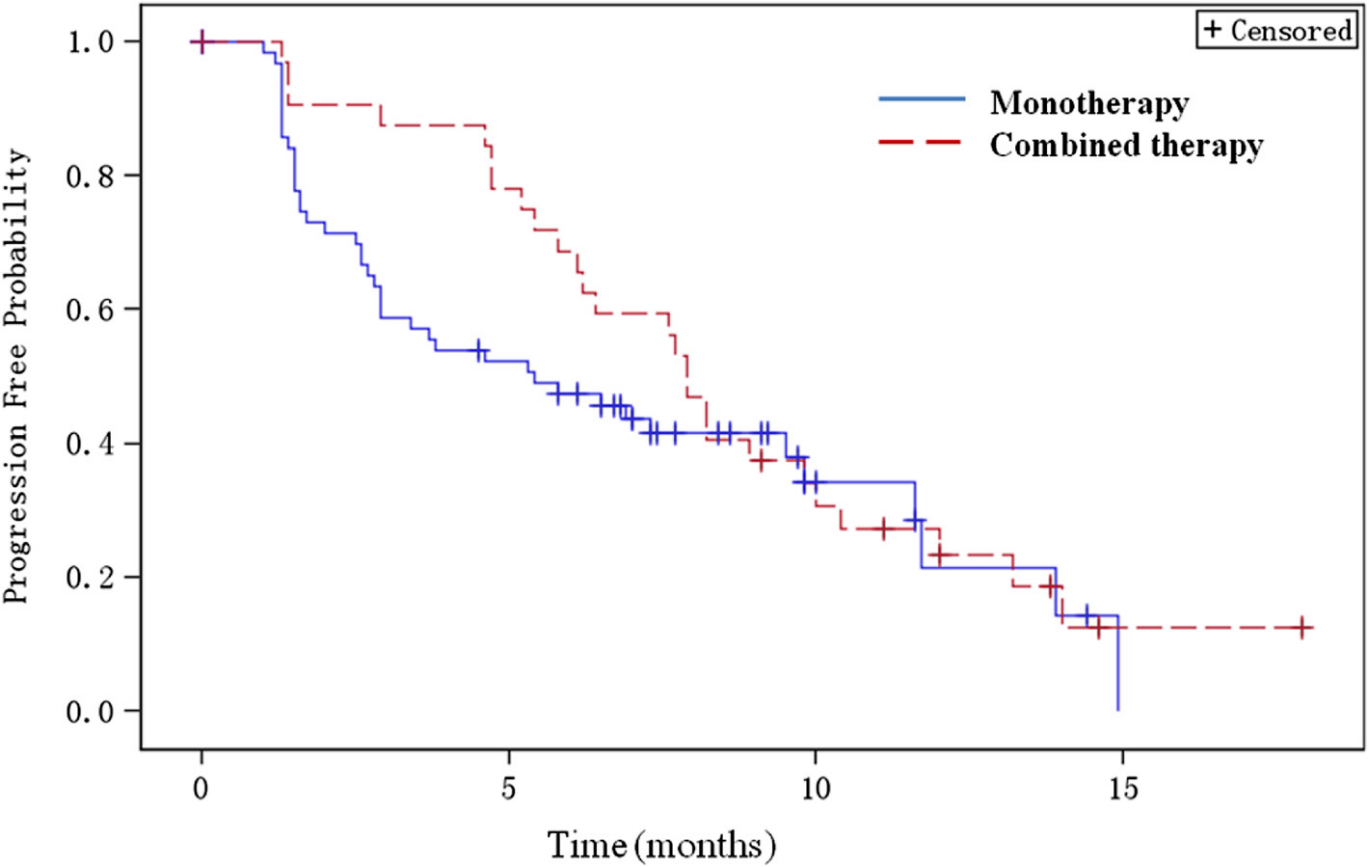
Kaplan-Meier survival estimates for PFS—full analysis set. Median PFS for the monotherapy and combined therapy groups was 5.40 months (95 % CI 2.90, 9.80) and 7.90 months (95 % CI 6.10, 9.80), respectively

In the combination therapy study, one study subject in the 30-mg/m^2^ dose group discontinued the study because of an AE (considered unlikely to be related to the study drug) before completing the first treatment cycle and was deemed not evaluable. Therefore, 32 combination therapy study subjects were evaluable for efficacy. The ORR was 42.4 % (FAS), which included one study subject who had CR and 13 study subjects who had PR (Table 3), 95 % CI (26.6 %, 60.9 %). Twenty-two (66.7 %, FAS) study subjects achieved response (CR or PR) or maintained stable disease for 6 months or longer. Of the 14 study subjects with CR or PR, three had received prior chemotherapies containing an anthracycline, a taxane, and capecitabine. Of the 15 study subjects with SD, six had received prior chemotherapies containing all three types of agents. By the study end date (Jan. 31, 2014) in terms of PFS, 26 (78.8 %) patients experienced disease progression. The median PFS (Fig. 2) was 7.9 months (FAS, 95 % CI = 6.1, 9.8), and the median duration of response was 7.8 months. Eighteen patients (54.5 %) had died by Jan. 23, 2016.

**Table 3:** Study subject end of treatment objective response rates

Utidelone starting dose, mg/m^2^	Best response,^a^ *n*						ORR,^b^ %	
	CR	PR	SD	PD	NA	Total	ORR	95 % CI^c^
Combination therapy: utidelone (30 mg/m^2^) and capecitabine (2000 mg/m^2^) *n* = 33								
25	0	2	1	0	0	3	66.7	9.4, 99.2
30	1	11	10	2	1	25	48.0	27.8, 68.7
35	0	0	4	1	0	5	0	–
Total	1	13	15	3	0	33	42.4	26.6, 60.9
Monotherapy: utidelone (40 mg/m^2^) *n* = 70								
40	1	19^d^	25	18	7	70	28.57	18.40, 40.62

*CR* complete response, *PR* partial response, *SD* stable disease, *PD* progressive disease, *ORR* objective response rate, *CI* confidence interval

^a^Assessed according to RECIST1.1

^b^ORR = (CR + PR)/total × 100 %

^c^Calculated using Clopper Pearson method

^d^Including 17 patients with PR and 2 patients with unconfirmed PR

In the monotherapy study, 63 (90 %) of the 70 enrolled study subjects completed at least 2 cycles of treatment and were therefore evaluable for efficacy. Of the seven study subjects not evaluable for efficacy, two discontinued during the first cycle due to SAEs and two terminated due to AEs after completing the first cycle, one left the study due to lung metastasis and severe cough and two others withdrew from the study for other reasons. Of the 63 study subjects evaluated, 20 completed 6 cycles of utidelone treatment, 26 discontinued before completing 6 cycles because of PD, and 17 did not complete 6 cycles because of AE or other reasons. By the end of the study, 1 study subject with CR, 19 with PR (including two with unconfirmed PR), and 25 with SD (Table 3) were observed. The ORR was 28.57 % (95 % CI 18.4, 40.6). The median duration of response was 7.4 months. Twenty-nine study subjects achieved clinical benefits, defined as maintaining stable disease or response (CR or PR) for at least 6 months. The median time to response was 6 weeks. By the study end date (Dec. 31, 2014), 45 (64.3 %) of the 70 enrolled patients had disease progression, including 6 who had died without observing PD. The median PFS (Fig. 1) for FAS was 5.4 months (95%CI, 2.9, 9.8). Thirty-three patients (47.1 %) had died by Jan. 23, 2016.

### Tolerability

The most common AEs reported in the combination therapy and monotherapy studies are summarized in Table 4 using NCI CTCAE version 3.0 grade criteria. In the combination therapy, 33 patients were evaluable for safety. No SAEs occurred or deaths resulted that were attributed to the study treatment. The major AE associated with uitdelone was peripheral neuropathy (PN), primarily classified as sensory. Grade 3 PN was reported for 45.5 % of the combination therapy study subjects and was managed with dose reduction, increasing dosing interval, treatment interruption, or symptomic management by adjunctive treatments. The majority of the study subjects continued dosing after recovering to grade 1 or better within 2 weeks. Other toxicities were as expected for this combination therapy, including hand-foot syndrome resulted from capecitabine, nausea, vomiting, myalgia and arthralgia, fatigue, and alopecia. Most of the AEs were grade 1 or 2 and were considered manageable and reversible.

**Table 4:** Study subject adverse events (AEs) by severity

AEs^a^	Number (%) patients					
Grade	Grade 1	Grade 2	Grade 3	Grade 1	Grade 2	Grade 3
Study	Combination therapy: utidelone (30 mg/m^2^) + capecitabine (2000 mg/m^2^) *n* = 33			Monotherapy utidelone (40 mg/m^2^) *n* = 70		
Event of grade 3 or above	19 (57.6)			13 (18.6)		
Hematologic toxicity	7 (24.2)	5 (15.2)	2 (6.1)	8(11.4)	9 (12.9)	5 (7.1)
Neutrophil decreased	2 (6.1)	2 (6.1)	2 (6.1)	11(15.7)	3(4.3)	5 (7.1)
WBC decreased	5 (15.2)	6 (18.2)	0	6 (8.6)	9 (12.9)	1(1.4)
Hemoglobin decreased	1 (3.0)	0	0	1 (1.4)	2(2.9)	0
Platelet decreased	0	2 (6.1)	0	2 (2.9)	0	0
Hepatic and renal function abnormalities	2 (6.1)	1 (3.0)	0	8 (8.6)	3 (4.3)	0
GGT increased	0	0	0	1 (1.4)	1 (1.4)	0
ALT increased	1 (3.0)	0	0	7 (10)	2( 2.9)	0
AST increased	1 (3.0)	0	0	4 (5.7)	2 (2.9)	0
Total bilirubin increased	1 (3.0)	1 (3.0)	0	3 (4.3)	0	0
Gastrointestinal toxicity	10 (30.3)	17 (51.5)	0	28 (40)	10 (14.3)	2 (2.9)
Decreased appetite	19 (57.6)	0	0	14 (20)	1 (2.9)	0
Diarrhea	8 (24.2)	2 (6.1)	0	9 (12.9)	5 (7.1)	2 (2.9)
Vomiting	4 (12.2)	10 (30.3)	0	6 (8.6)	1 (1.4)	0
Nausea	10 (30.3)	16 (48.5)	0	22 (31.4)	2 (2.9)	0
Neurological toxicity	2 (12.2)	13 (39.4)	17 (51.5)	28 (40)	23 (32.9)	7 (10)
Peripheral neuropathy	4 (12.2)	13 (39.4)	15 (45.5)	26 (37.1)	23 (32.9)	6 (8.6)
Insomnia	10 (30.3)	0	1 (3.0)	5 (7.1)	0	0
Dizziness	17 (51.5)	1 (3.0)	2 (6.1)	7 (10)	4 (5.7)	2 (2.9)
Hand-foot syndrome	7 (21.2)	1 (3.0)	5 (15.2)	1 (1.4)	0	0
Other						
Myalgia and arthralgia	5 (15.2)	15 (45.5)	5 (15.2)	9 (12.9)	1 (1.4)	1 (1.4)
Alopecia	2 (6.1)	4 (12.2)	1 (3.0)	20 (28.6)	4 (5.7)	1 (1.4)
Fatigue	13 (39.4)	6 (18.2)	1 (3.0)	8 (11.4)	9 (12.9)	5 (7.1)

^a^AEs according to Common Terminology Criteria for Adverse Events version 3.0

In the monotherapy study, 70 patients were evaluated for safety. The major AE related to utidelone was PN (78.6 % for all grades), most of which were grades 1 and 2 with six study subjects experiencing grade 3 PN (8.6 %). Of the 29 study subjects experiencing grade 2 or 3 PN, the median time to recovery was 18 days. All study subjects with severe PN (grade 3) experienced symptom resolution to grade 1 or less following cessation of utidelone administration. Other frequent AEs included short-term myalgia, arthralgia, nausea, vomiting, and loss of appetite. All AEs were considered manageable. Neutropenia occurred in 19 (27.1 %) of the 70 study subjects in the monotherapy study. Five study subjects (7.1 %) in the monotherapy study experienced grade 3 neutropenia; however, two were considered abnormal at study onset, prior to administration of the first treatment.

Two SAEs occurred during the monotherapy study, one study subject experienced a grade 3 neurological toxicity during cycle 1, presenting with headache and peripheral neuropathy that led to hospitalization. Symptoms were resolved and the study subject was recovered 3 weeks after discontinuation of utidelone treatment. Another study subject experienced myocardial ischemia after cycle 1, which resulted in prolonged hospitalization. Both SAEs were considered probably related to utidelone administration.

### Laboratory tests, vital signs, and physical examination

In general, very limited and mild myelosuppression toxicity was observed in both monotherapy and combination studies, indicating a very unique and advantageous feature of utidelone compared with ixabepilone and taxanes. In addition, abnormal liver and renal functions were not frequently found, suggesting utidelone treatment, under conditions of these studies, was unlikely to result in significant adverse liver and renal function effects. Moreover, diarrhea was not observed frequently, implicating that the gastrointestinal toxic effect of utidelone is quite mild. Other observed toxicities were consistent with the common adverse events of chemotherapy agents. Two study subjects had abnormal blood glucose levels, which were deemed unlikely to be related to the study treatment. Clinically significant abnormal levels of serum creatinine and blood urea nitrogen were not observed. Abnormal clinical laboratory test measurements reported for any study subjects during the course of treatment were not considered severe enough to require dose adjustment or treatment interruption. Clinically significant abnormalities or changes from baseline were not observed in ECG, urinalysis, ECOG performance status, body weight, or other vital signs for study subjects during the course of the study treatments.

## Discussion

In the treatment of metastatic breast cancer, tumor resistance to the standard chemotherapy or adjuvant chemotherapy with anthracyclines and taxanes remains an area of substantial unmet medical needs.

The antineoplastic activities of epothilones have been well characterized in vitro and in vivo and further established by the FDA approval of the epothilone analog, ixabepilone [1–4, 7, 8]. In clinical trials, ixabepilone has demonstrated efficacy in the treatment of metastatic and locally advanced breast cancer in patients who had gone through multiple courses of standard chemotherapies and had failed or suffered recurrence [4, 9–11]. However, the toxicities related to ixabepilone treatment, such as neutropenia (grade 3, 31 %; grade 4, 23 %), sensory neuropathy (PN, grade 3, 13 %; grade 4, 1 %), and fatigue (grade 3, 13 %; grade 4, 1 %) were very prominent and often resulted in discontinuation of the treatment.

Utidelone is an epothilone analog produced by genetically engineered *S. cellulosum* using enhanced production and fermentation technology [6]. The active ingredient is isolated and purified directly from the fermentation of the engineered production strain without further chemical synthesis or modification. Thus, the highly purified utidelone can be manufactured with well-controlled quality and scalable production capacity. This process leads to high yields and a low cost of utidelone, in contrast to the first FDA-approved epothilone drug ixabepilone, which needs several steps of chemical synthesis after bacterial fermentation and had higher cost. The high incidence of grade 3 and grade 4 toxicities, together with the high cost of ixabepilone, had greatly hindered the clinical use of this drug.

This report of two open-label studies of utidelone in metastatic breast cancer patients refractory to anthracyclines and/or taxanes, demonstrated a favorable efficacy result, with once daily for 5-day dose regimen instead of the standard q3w treatment. The combination therapy study showed an ORR of 42.4 %, a median PFS of 7.9 months, and a median duration of response of 7.8 months. On the other hand, the utidelone monotherapy study resulted in a 28.57 % ORR, a 5.4-month median PFS, and a 7.3-month median duration of response. By Jan. 2016, 18 patients had died in the combination study arm and 14 patients still survived; the preliminary median overall survival (OS) was 30.6 months. In the monotherapy arm, 33 of the 70 patients had died, 27 patients still survived, and 10 were lost to follow-up; the preliminary median OS was 21.2 months. These preliminary data suggested that utidelone may also significantly increase the OS either used as a single agent or in combination with capecitabine. However, the final OS still needs follow-up and remains to be determined. In comparison, the phase III combination therapy trial of ixabepilone plus capecitabine in metastatic and locally advanced breast cancer patients resulted in a 34.7 % ORR, a 5.7-month median PFS, and a 6.4-month median duration of response [4]. However, ixabepilone plus capecitabine did not improve OS even for the patients who received two or fewer prior chemotherapy regimens [12] and less pre-treated than the patients enrolled in our study. The phase II ixabepilone monotherapy trial study in patients with taxane-resistant MBC reported a 12 % ORR, a 2.2-month median PFS, and a 10.4-month median duration of response [13]. Although the current utidelone studies had smaller sample sizes than the reported ixabepilone studies, our findings suggest that utidelone may have efficacy more favorable to that of ixabepilone, including ORR, PFS, and possibly also OS. These results also indicated that utidelone could overcome resistance to anthracyclines and taxanes, providing a novel therapeutic option to fight against multidrug-resistant breast cancer. The mechanisms underlying utidelone to overcome drug resistance are yet to be investigated. It had been reported previously that epothilone was not a substrate for p-glycoprotein, also known as multidrug-resistant protein 1 (MDR1), or breast cancer resistance protein, also known as BCRP/ABCG2 [14, 15].That may confer an advantage to this investigational drug to bypass the mechanisms of drug resistance. Utidelone may also gain the ability to overcome drug resistance via other mechanisms. In this regard, it had been reported that epothilones could inhibit the functions of actin cytoskeleton and its critical regulator Rac1 GTPase [16], which are important players in multidrug resistance [17–19].

In both combination and monotherapy studies, the utidelone treatment-related toxicities were generally mild to moderate and considered clinically manageable. The major AEs associated with administration of utidelone alone or in combination with capecitabine were PN, myalgia, and arthralgia. Of these associated AEs, even though PN was considered to be the most problematic, it could be managed with dose delay, dose reduction, or treatment discontinuation, generally resulting in recovery within 14–18 days for grade 3 PN. There were no deaths attributed to utidelone administration during both studies. Overall, utidelone was considered well tolerated in these phase II trials.

In comparison with published data of other chemotherapies with similar mechanism of action, especially with ixabepilone, utidelone showed some unique and important features in terms of safety profile. One surprise to the researchers was that the incidences of neutropenia and other hematological toxicities were much less prominent, suggesting that utidelone has quite mild effect on myelosuppression, in contrast to paclitaxel, and ixabepilone which in combination with capecitabine, as high as 70 % of patients had grade 3/grade 4 neutropenia. It seemed that the PN effect of utidelone, although prominent, may also not so severe as ixabepilone considering the incidence of grade 3/grade4 events, which was less with much shorter recovery time to grade 1 or baseline under our study conditions than that of ixabepilone [4]. In addition, utidelone did not appear to have clinical significant effects on hepatic and renal functions. Moreover, the gastrointestinal toxic effect of utidelone seemed to be very mild, since diarrhea and vomiting was not observed frequently, but some other epothilone analogs, such as epothilone B, caused severe gastrointestinal toxicities [20]. Although the mechanism underlying these differences in toxic effects is yet to be elucidated, these unique safety features represent important advantages of utidelone.

## Conclusions

In conclusion, utidelone demonstrated clear efficacy with significant PFS improvement, good tolerability, and manageable adverse events with an advantageous toxicity profile. This study strongly supports that utidelone is a promising novel investigational drug as a single agent and in combination with capecitabine as well for the treatment of patients with advanced metastatic breast cancer who had failed multiple courses of chemotherapy including anthracyclines and taxanes or suffered recurrence. Utidelone could also offer an important therapeutic option in the armamentarium of cancer treatment, especially for cancers that are multidrug resistant.

## Abbreviations

CR, complete response; CTC, common toxicity criteria; ECOG, Eastern Cooperative Oncology Group; FDA, Food and Drug Administration; NCI, National Cancer Institute; ORR, objective response rate; OS, overall survival; PFS, progression-free survival; PN, peripheral neuropathy; PR, partial response; RECIST, Response Evaluation Criteria in Solid Tumors; SD, stable disease; UTD1, utidelone
